# Nitrogen Regulator GlnR Controls Redox Sensing and Lipids Anabolism by Directly Activating the *whiB3* in *Mycobacterium smegmatis*

**DOI:** 10.3389/fmicb.2019.00074

**Published:** 2019-01-29

**Authors:** Di You, Ying Xu, Bin-Cheng Yin, Bang-Ce Ye

**Affiliations:** ^1^Laboratory of Biosystems and Microanalysis, State Key Laboratory of Bioreactor Engineering, East China University of Science and Technology, Shanghai, China; ^2^Institute of Engineering Biology and Health, Collaborative Innovation Center of Yangtze River Delta Region Green Pharmaceuticals, College of Pharmaceutical Sciences, Zhejiang University of Technology, Hangzhou, China

**Keywords:** *Mycobacterium*, GlnR, WhiB3, nitrogen metabolism, SL-1, redox stress

## Abstract

WhiB3 is a conserved cytoplasmic redox sensor which is required in the infection and lipid anabolism of *Mycobacterium tuberculosis*. The response of WhiB3 to environmental nutrient and its regulatory cascades are crucial during the persistent infection, while little is known about the relationship between WhiB3 and emergence of nutrient stress in this process. Here, we found that nitrogen regulator GlnR directly interacted with the WhiB3 promoter region and activated its transcription in response to nitrogen availability. In *whiB3* promoter region, the typical GlnR-box was also identified. Moreover, GlnR controlled cell resistance to redox stress and SL-1 lipid anabolism by directly activating *whiB3* expression. These results demonstrated that GlnR regulated redox sensor WhiB3 at the transcriptional level and mediated the interplay among nitrogen metabolism, redox sensing, and lipid anabolism.

## Importance

The regulatory network of mycobacteria has delicately evolved to adapt to the extreme nutrient deficiencies and various stress including acidic stress and redox stress during infection. Identification the roles of critical members that participate in this network is crucial to the development of novel clinical therapies. Here, we found that WhiB3, a redox-sensing regulator, was under direct transcription activation of nitrogen regulator GlnR during nitrogen starvation. In addition, GlnR controlled cell resistance to redox/acidic stress and SL-1 lipid anabolism through WhiB3, which suggested that the GlnR-mediated WhiB3 regulatory pathway might influence mycobacterial infection. These findings provide novel insight into the tight connection of intracellular redox status, potential virulence, and nitrogen metabolism.

## Introduction

Tuberculosis is one of the major global health emergency, whose pathogenesis is closely related to its cell wall lipid (Dye et al., 1999). The enzymes and regulators involved in the lipid synthesis and regulation are typically ideal drug targets due to their uniqueness (Brennan and Nikaido, 1995; Kolattukudy et al., 1997; Barry et al., 1998; Daffe and Draper, 1998), the regulatory protein WhiB3 was one of which has been well characterized associating with redox sensing and lipid anabolism in *Mycobacterium tuberculosis* during infection (Singh et al., 2009).

WhiB3 is a member of the WhiB subfamily with the common characteristic of small molecular weight and high cysteine content (Flardh et al., 1999). WhiB protein is initially found in *Actinobacteria* (Chater, 1972) and predicted to be important transcriptional factor participated in pathogenesis, cell division, and other stresses sensing. *M. tuberculosis* genome contains seven annotated *whiB* genes (Alam et al., 2009), elucidation of their functions and regulatory network in mycobacteria is beneficial for understanding the pathogen biology and finding out appropriate countermeasure. Previous studies had shown that *M. tuberculosis* WhiB3 controlled virulence in two animal models (Steyn et al., 2002), and the WhiB orthologs were implicated in varieties of pathways including sporulation, cell division (Gomez and Bishai, 2000), oxidative stress (Kim et al., 2005), pathogenesis, and antibiotic resistance (Morris et al., 2005). During infection, *M. tuberculosis whiB3* expression was significantly induced (Rohde et al., 2007). The *whiB3* deficiency strain exhibited a pathology defect, altered colony rugosity, and growth properties (Steyn et al., 2002; Singh et al., 2007). WhiB3 was also involved in maintaining intracellular redox homeostasis through regulating the fatty acids metabolisms via a redox switching mechanism (Singh et al., 2009; Cumming et al., 2017). *M. tuberculosis* WhiB3 was identified as a regulator of virulence lipid anabolism and distinctively modulated the propionate assimilation into complex virulence lipids including polyacyltrehalose (PAT), sulfolipid (SL-1), phthiocerol dimycocerosate (PDIM), and storage lipid triacylglycerol (TAG) under defined oxidizing/reducing conditions.

*Mycobacterium smegmatis* is a fast-growing non-pathogenic species generally used as a model for slow-growing pathogenic *M. tuberculosis*. In *M. smegmatis*, the WhiB subfamily protein WhmD (homologous to WhiB2 in *M. tuberculosis*) was an essential gene necessary in proper septation and cell division (Raghunand and Bishai, 2006). Moreover, during nutrient starvation, WhiB2, WhiB3, and WhiB4 participated in the development of both mono-nucleoided small resting cells and log-phase-sized resting cells with similar temporal expression pattern (Wu et al., 2016). Differing from the well-studied function of WhiB3, the regulatory factors and mechanisms acted on WhiB3 is still little known except that its DNA binding ability is regulated by a thiol-disulfide redox switch reversibly (Singh et al., 2009), it is a downstream target of RelA (Primm et al., 2000; Rifat et al., 2009), and it interacts with σ factor RpoV (Steyn et al., 2002).

In the present study, we investigated a notable role of nitrogen regulator GlnR in regulating *whiB3* transcription responding to nitrogen availability of *M. smegmatis*. We found out that GlnR directly bound with *whiB3* promoter region and activated *whiB3* transcription during nitrogen starvation. Moreover, GlnR controlled the cell resistance to redox stress and SL-1 lipid anabolism by directly activating the expression of *whiB3*. These results elucidated the mechanism of GlnR-mediated interplay between nitrogen metabolism and stress sensing, which possibly contributed toward understanding pathogen biology of mycobacteria.

## Materials and Methods

### Strains, Plasmids, and Growth Conditions

Strains and plasmids used in this study were listed in Supplementary Table S1. *E. coli* DH5α (TransGen Biotech) was used for cloning and cultured in LB media supplemented with 50 μg/ml kanamycin. *M. smegmatis* MC^2^ 155 WT, Δ*glnR* and complemented strains were cultured in LB media supplemented with 0.05% tween 80 or Sauton’s medium. Nitrogen free Sauton’s medium (0.05% (*w*/*v*) MgSO_4_, 0.05% (*w*/*v*) KH_2_PO_4_, 0.2% (*w*/*v*) citric acid, 0.2% (*v*/*v*) glycerol, 0.005% (*w*/*v*) ferric citrate, 0.015% (*v*/*v*) Tyloxapol, 0.0001% (*v*/*v*) ZnSO_4_) supplemented with 1 mM (nitrogen-limited, N^L^) or 30 mM (nitrogen-rich, N^XS^) (NH_4_)_2_SO_4_ was used for the assessment of transcription level (Jenkins et al., 2013).

### Overproduction and Purification of GlnR and WhiB3 Proteins

The overproduction and purification of His-tag GlnR protein was performed as previously described (Xu et al., 2017). The *whiB3* gene was amplified from *M. smegmatis* with primers listed in Supplementary Table S2. The purified PCR product using the PCR purification kit (TransGen Biotech) was cloned into pET-28a (+), and generated the recombinant plasmid pET-*whiB3.* The single colony was grown overnight in 5 mL LB medium containing 50 μg/ml kanamycin at 37°C. Then transferred into 100 mL LB medium until the OD_600_ reached 0.6 and induced with 0.5 mM IPTG. The His-WhiB3 protein was purified from soluble fraction performed as described (Singh et al., 2009; You et al., 2014). Apo-WhiB3 protein was prepared as described previously (Singh et al., 2009), the purified apo-WhiB3 protein was then used for *in vitro* EMSA experiments.

### Electrophoretic Mobility Shift Assays (EMSAs)

The upstream 350 bp region of *whiB3* gene containing the putative GlnR-box was amplified with primers listed in Supplementary Table S2. PCR products were labeled with biotin using the universal biotinylated primer (5’-AGCCAGTGGCGATAAG-3’). The EMSA probes were purified using PCR purification kit (TransGen Biotech) and analyzed by agarose gel electrophoresis. The probes concentration was determined with microplate reader (BioTek, United States). EMSA assays were carried out with the Chemiluminescent EMSA Kit (Beyotime Biotechnology, China). After incubation at 25°C for 20 min, samples were loaded and separated on 6% non-denaturing PAGE gel in ice-cold 0.5% Tris-borate-EDTA at 100 V and determined by BeyoECL Plus.

### RNA Preparation and RT-PCR

*Mycobacterium smegmatis* MC2 155 and its mutant strains were activated in LB medium (containing 0.05% tween 80) for 36–48 h at 37°C and then transferred into N^XS^ and N^L^ Sauton’s medium. Mid-exponential cells of *M. smegmatis* were collected by centrifugation (12000 rpm, 5 min, 4°C). Total RNA was obtained with the RNAprep Pure Cell/Bacteria kit (Tiangen Biotech, Beijing, China). The quality of RNA was analyzed by electrophoresis. The RNA concentrations were determined with microplate reader (BioTek). 1 μg RNA was used as template for cDNA synthesis with the PrimeScript reverse transcription (RT) regent kit (TaKaRa, Japan). The genomic DNA was removed before reverse transcription by DNase digestion for 5 min at 42°C. RT-PCR experiments were conducted with SYBR Premix Ex Taq GC kit (TaKaRa, Japan), using the primers listed in Supplementary Table S2. PCR assays were carried out as described previously (You et al., 2017).

### Construction of *whiB3*^MU^ Strain

**FIGURE 1:**
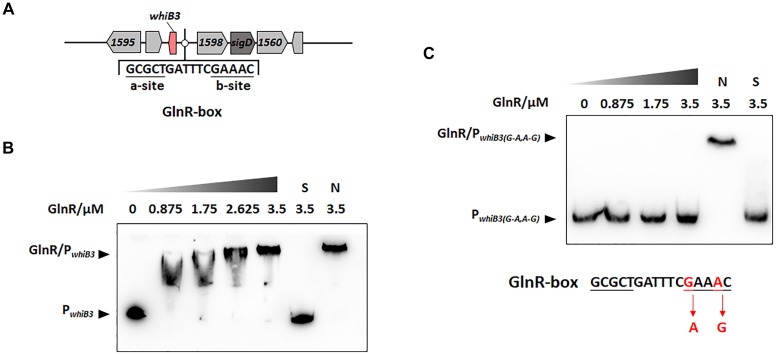
GlnR binding with the upstream promoter region of *whiB3* in *M. smegmatis*. **(A)** GlnR-binding site (GlnR-box) in *whiB3* promoter region. **(B)** EMSA of purified GlnR with the *whiB3* promoter region. 10 nM DNA probes were incubated with a gradient concentration of purified GlnR. **(C)** EMSAs of purified GlnR protein with mutated *whiB3* promoter regions.

A *whiB3*^MU^ mutant was generated by electroporation with recombined plasmid pPR27 (Pelicic et al., 1997) which consisted of the mutated GlnR-box (consistent with the Figure 1C) and kanamycin resistance. The recombination cassette was obtained through homologous recombination and confirmed by PCR analysis and DNA sequencing. After the above cloning steps, the recombination cassette was transferred into the suicide plasmid pPR27 to generate final plasmid pPR27-*whiB3*^MU^ and then introduced into *M. smegmatis* strains by electroporation. The selected mutants were further confirmed by PCR analysis and DNA sequencing.

### Overexpression of WhiB3 in *M. smegmatis*

With *M. smegmatis* genomic DNA as template, *whib3* was amplified with the primers listed in Supplementary Table S2. Purified PCR products were digested with BamHI and HindIII, then inserted into the corresponding sites of integrative plasmid pMV261 previously digested with the same enzymes. By electroporation transformation (Manganelli et al., 2001), the plasmid was introduced into *M. smegmatis* strains (wild type and Δ*glnR*). The final overexpression strains were screened using kanamycin resistance and confirmed by PCR with primers PMV-F and PMV-R (Supplementary Table S2).

### CFU Colony Count

*Mycobacterium smegmatis* WT, Δ*glnR*, Δ*glnR*::*glnR* and Δ*glnR*::*whiB3* strains were inoculated in 5 mL LB medium with Tween for approximately 48 h. Then transferred into N^L^ Sauton’s medium with different pH and different H_2_O_2_ concentrations at 37°C for 24 h. Cultures unexposed to acid or H_2_O_2_ were used as controls. The exposed/unexposed cultures were then diluted for bacterial viability at the indicated time points.

### Construction of the *whiB3-lacZ*^+^ Reporters

DNA fragments containing the *lacZ* gene from *Escherichia coli* (*E. coli* str. K-12 substr. MG1655) were amplified by PCR assays using lacZ-F-O and H-HindIII-lacZ-R primers (Supplementary Table S2). The promoter of *whiB3* gene was amplified using the H-KpnI-1597P-F and 1597P-R-O primers (Supplementary Table S2). The two pairs of primers contained an overlap sequence. Using the above PCR products as template, H-KpnI-1597P-F and H-HindIII-lacZ-R as primers, *lacZ* gene and *whiB3* promoter were linked together. The PCR fragment was then cloned into plasmid pMV261 (Stover et al., 1991). The recombinant plasmid was introduced into *M. smegmatis* strains by transformation of electroporation (Manganelli et al., 2001). The *whiB3-lacZ^+^* reporter with kanamycin resistance was selected for subsequent experiments.

### *In vitro*β-Galactosidase Assays

*In vitro*β-Galactosidase activity was analyzed using previous methods with some modifications. The strains contain lacZ were cultured in LB medium supplemented with 0.05% tween 80 and Sauton’s medium until the exponential phase. 10 μL cells were added into 990 μL Z buffer (40 mM NaH_2_PO_4_, 6 mM Na_2_HPO_4_, 10 mM KCl, 50 mM β-mercaptoethanol, 1 mM MgSO_4_). The mixtures were incubated for 15 min at room temperature. 200 μL substrate O-nitrophenyl-β-D-galactosidase (ONPG, 4 mg/mL in 100 mM KH_2_PO_4_, pH 7.0) was added, and the reaction was stopped with the addition of 0.2 mL 2.5 mM Na_2_CO_3_. Optical density of the solution at 420 nm was then measured. β-galactosidase activity in modified Miller units was calculated as following: (OD_420_ × 1000)/(*t* ×*V* × OD_600_), in which “*V*” represents the volume of culture used in milliliters and “*t*” represents the incubation time in minutes.

### Polar Lipid Extraction and TLC Analysis

Lipids extraction, fractionation and analysis were performed as previously described (Jackson et al., 1999). The *M. smegmatis* WT and WhiB3 overexpression strain (O*whiB3*) were activated in 5 mL LB medium with Tween for approximately 48 h. Then transferred into N^L^ Sauton’s medium for 24 h. The bacteria were centrifuged at 12,000 × g for 10 min for several times to collect 50 mg bacteria (wet weight). Bacteria were extracted first with CHCl_3_/CH_3_OH (1:2, *v*/*v*), and then with CHCl_3_/CH_3_OH (2:1, *v*/*v*). After centrifugation, polar lipids were extracted in the supernatant. The dried lipid extracts were analyzed using chloroform: ethanol: water (90:10:1) by TLC. Glycolipid spots were visualized by spraying anthrone (0.2% in sulfuric acid), and followed with charring at 115°C. The band intensities were quantified by densitometry with Image J software using the Substract Background option.

## Results

### Nitrogen Response Regulator GlnR Directly Activated *whiB3* Gene in *M. smegmatis*

The DNA binding sites of GlnR (GlnR-box) in actinobacteria (Yao et al., 2014) and *Mycobacteria* (Xu et al., 2017; Liu et al., 2018) were identified in previous studies, the putative GlnR-box was identified in the upstream region of *whiB3* gene in *M. smegmatis*, consisting of a-site and b-site separated with six nucleotides (a-site-n6-b-site) (Figure 1A). To investigate whether GlnR could directly bind to the *whiB3* upstream region, EMSA was performed. A 200-fold excess of unlabeled specific probes (S) and non-specific competitor DNA (sperm DNA) (N) were used as controls. The results in Figure 1B showed obvious shift bands following incubation with purified His-tag GlnR, suggesting that GlnR bound to the promoter region of *whiB3*. A mutation of GlnR-box was constructed (G mutated to A, A mutated to G in the conserved site of b-site shown in red, Figure 1C) and then subjected to EMSA assay. EMSA revealed that the mutation inhibited GlnR binding with *whiB3* promoter region (Figure 1C). The result indicated that these nucleotides in the b-site are essential for binding of GlnR to the *whiB3* promoter region.

**FIGURE 2:**
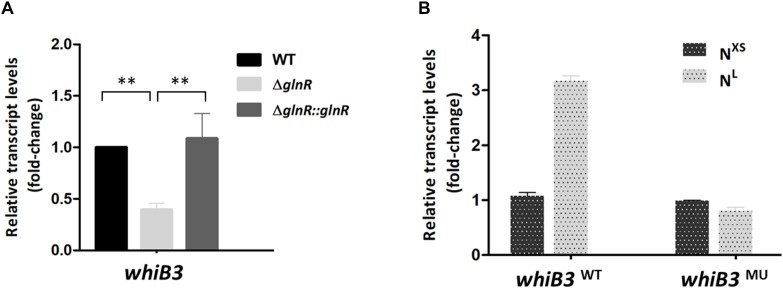
The *whiB3* expression is directly controlled by GlnR. **(A)** The *whiB3* transcription levels in *Mycobacterium smegmatis* WT, Δ*glnR* and Δ*glnR*::*glnR* strains grown in N^L^ medium till the middle exponential phase. Fold change represented the expression level compared to the *whiB3* expression in WT strain. **(B)** The *whiB3* transcription levels in *M. smegmatis whiB3*^WT^ and *whiB3*^MU^ strains grown in N^XS^/N^L^ condition. Fold change represented the *whiB3* transcription level compared to *whiB3*^WT^ strains grown in the N^XS^ condition. The error bars showed standard deviations from three independent experiments. ^∗∗^*P* < 0.01.

We then examined whether GlnR has regulatory effect on *whiB3* using *M. smegmatis glnR*-deletion strain (Δ*glnR*) and *glnR* complemented strain (Δ*glnR*::*glnR*) constructed as described previously (Xu et al., 2017; Liu et al., 2018). During growth in N^L^ medium, the transcription level of *whiB3* in *M. smegmatis* wild type (WT), Δ*glnR* and complemented strains were detected. As shown in Figure 2A, *glnR* deficiency caused a 70% decrease in *whiB3* transcription compared with the WT strain, and was restored in the *glnR* complemented strain. Thus, GlnR directly activated the transcription of *whiB3* gene in *M. smegmatis*.

### Intracellular *whiB3* Expression Is Influenced by Nitrogen Availability

The transcription response of *whiB3* gene to nitrogen signals was then investigated. Using homologous replacement, we constructed a mutant strain (*whiB3*^MU^) with a mutant GlnR-box (GCGCTGATTTC**A**AA**G**C) in the *whiB3* promoter region as used in the EMSA experiment above (Figure 1C). To confirm GlnR is necessary in the *whiB3* expression changes influenced by nitrogen, *whiB3* with wild type GlnR-box (*whiB3*^WT^) was used as control. The two strains were grown in N^XS^ or N^L^ media. As shown in Figure 2B, in the *whiB3*^WT^ strain, the nitrogen starvation resulted in a 3-fold higher *whiB3* expression compared to the level in N^XS^ media. While in the *whiB3*^MU^ strain, *whiB3* transcription showed almost no response to nitrogen signals. EMSA result also revealed that GlnR did not bind with the mutant GlnR-box *in vitro* (Figure 1C). Taken together, these observations demonstrated that *whiB3* expression was influenced by GlnR-mediated nitrogen availability in *M. smegmatis*.

### *WhiB3* Is Responsible for Response to Acidic and Redox Stress

**FIGURE 3:**
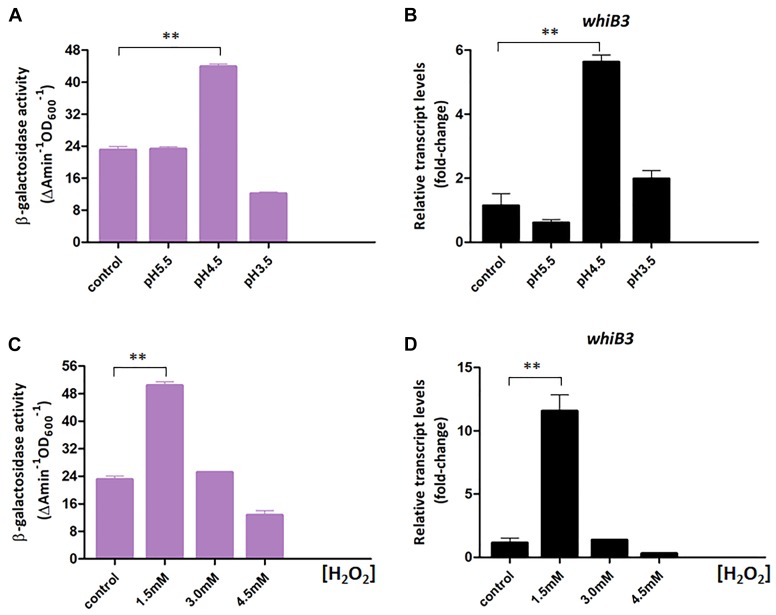
WhiB3 is responsible for response to environmental stress. **(A)** The β-galactosidase activity of *M. smegmatis* WT strain under different pH conditions. **(B)** The expression of *whiB3* under different pH conditions. **(C)** The β-galactosidase activity of *M. smegmatis* WT strain under different H_2_O_2_ concentrations. **(D)** The expression of *whiB3* under different H_2_O_2_ concentrations. Error bars represented standard deviations of three independent experiments. ^∗∗^*P* < 0.01.

In *Mycobacterium tuberculosis*, WhiB3 is reported necessary for maintaining redox homeostasis (Singh et al., 2009). Here we constructed a chromosomal *whiB3-lacZ^+^* reporter to monitor the *whiB3* transcription changes to pH and redox stress in *M. smegmatis*. The *whiB3-lacZ^+^* reporter strain was confirmed by PCR and SDS-PAGE analysis, then screened by β-galactosidase activity, leading to the final identification of *whiB3-lacZ^+^*-4 strain (Supplementary Figure S1). The β-galactosidase activity was used to reflect *whiB3* expression level accordingly. The reporter strain was cultured in Sauton’s medium for 24 h and transferred to fresh Sauton’s medium with different pH or H_2_O_2_ concentration prior to exponential phase. The strains transferred directly to Sauton’s medium with pH 7.0 or containing 0 mM H_2_O_2_ were used as controls, respectively. β-galactosidase activity in the *M. smegmatis* reporter strain was measured under different pH and H_2_O_2_ concentration, it achieved ∼5.5-fold higher at pH 4.5 than the control strain, and 10-fold higher when grown in 1.5 mM H_2_O_2_ concentration (Figure 3A,C). Meanwhile, the RT-PCR results displayed a relatively consistent trend of *whiB3* expression in response to different environment stress (Figure 3B,D). According to these data, we concluded that oxidative stress and acidic (low pH) conditions induced WhiB3 expression in *M. smegmatis.*

### *M. smegmatis* Responses to Environmental Stress Are Influenced by Lack of GlnR

**FIGURE 4:**
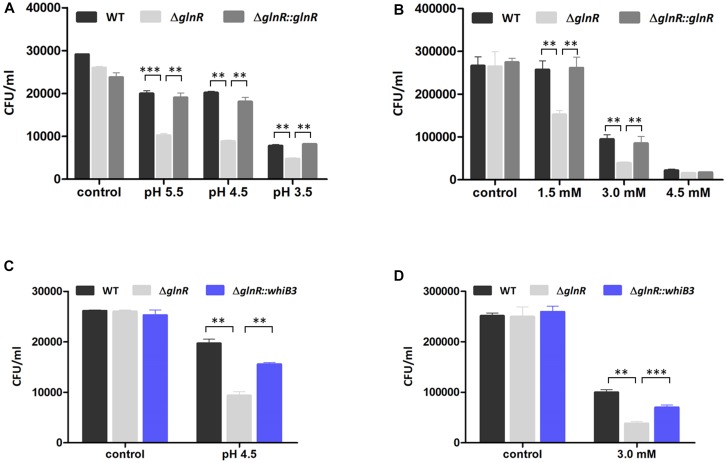
GlnR influenced the resistance of *M. smegmatis* to acidic and oxidative stress. **(A)** Effect of different pH condition on survival of WT, Δ*glnR* and Δ*glnR*-complemented strains grown in N^L^ Sauton’s medium. **(B)** Effect of different H_2_O_2_ concentration on survival of *M. smegmatis* strains grown in N^L^ Sauton’s medium. **(C)** Survival of WT, Δ*glnR* and Δ*glnR*::*whib3* strains grown in N^L^ Sauton’s medium with pH 4.5. **(D)** Survival of WT, Δ*glnR* and Δ*glnR*::*whib3* strains grown in N^L^ Sauton’s medium with 3 mM H_2_O_2_. Error bars showed standard deviations from three independent experiments. ^∗∗^*P* < 0.01, ^∗∗∗^*P* < 0.001.

The GlnR-mediated WhiB3 was responsible for the response to redox and acidic stress, raising a possibility that GlnR activation of *M. smegmatis* grown in N^L^ condition might exert an impact on the response to redox and acidic stress. We thus investigated whether GlnR influenced *M. smegmatis* growth under stress exposures via comparing the survival of WT, Δ*glnR,* and Δ*glnR*::*glnR* cells in different pH and H_2_O_2_ concentrations. The *M. smegmatis* WT, Δ*glnR* and Δ*glnR*::*glnR* strains were first activated in LB medium and then transferred into N^L^ Sauton’s medium with different pH or different H_2_O_2_ concentration, respectively. After 24 h incubation, the CFU was determined. As shown in Figure 4A,B, Δ*glnR* strain showed higher sensitivity to redox and acidic stress, the cell viability has a ∼2.4-fold decrease at pH 4.5 and ∼2.5-fold decrease in 3 mM H_2_O_2_. The resistance was substantially restored in the Δ*glnR*-complemented strain. When grown under pH 4.5 or 3 mM H_2_O_2_ condition, respectively, the most obvious differences in response to redox and acidic stress were observed (Figure 4A,B). The growth defect of Δ*glnR* strain under redox and acidic stress indicated that the phenotype might due to decrease of *whiB3* expression in Δ*glnR* strain. Next, we examined the cell survival of Δ*glnR* strain overexpressing WhiB3 (Δ*glnR*::*whiB3*) under pH 4.5 or 3 mM H_2_O_2_ condition. As shown in Figure 4C,D, the peroxide and acidic stress sensitivity of Δ*glnR* was substantially restored after overexpression of *whib3*, suggesting that the peroxide and acidity resistance was mainly contributed by GlnR-mediated activation of *whiB3*.

### The Transcription of Polyketide Synthase (MSMEG_4727, *pks5*) Is Influenced by GlnR-Mediated Nitrogen Availability

**FIGURE 5:**
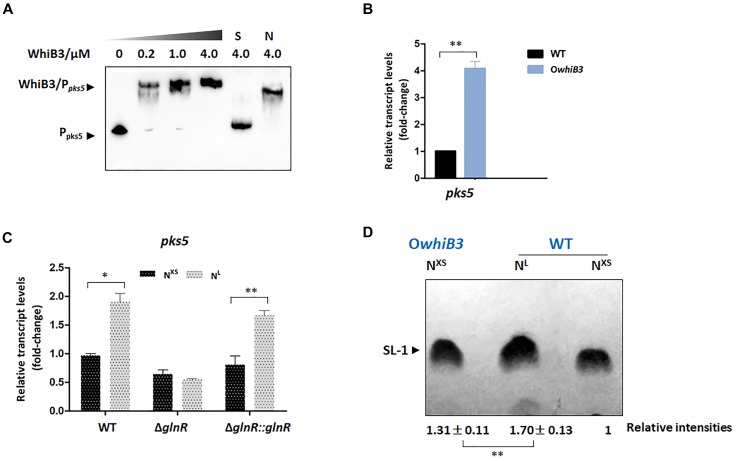
The biosynthesis of SL-1 lipid is regulated by GlnR-mediated WhiB3. **(A)** EMSA of apo-WhiB3 protein with *pks5* promoter region. A concentration gradient of apo-WhiB3 was incubated with 10 nM DNA probes. **(B)** The *pks5* transcript levels in *M. smegmatis* WT and O*whiB3* strains grown in N^L^ medium till the middle exponential phase. Fold changes represented the expression level compared to the *pks5* expression of WT. **(C)** The *pks5* transcript levels in *M. smegmatis* WT, Δ*glnR* and Δ*glnR*::*glnR* strains grown in N^XS^/N^L^ Sauton’s medium. Fold changes represented the *pks5* transcript levels compared to the *pks5* expression in N^XS^ condition. **(D)** *M. smegmatis* WT and O*whiB3* strains grown in N^XS^/N^L^ Sauton’s medium. In each lane, silica TLC plates were loaded with equal amount of polar lipids and developed in chloroform: ethanol: water (90:10:1). The band intensities were quantified by densitometry using Image J software. Relative band intensities were shown. Error bars showed standard deviations from three independent experiments. ^∗^*P* < 0.05, ^∗∗^*P* < 0.01.

WhiB3 was identified as a physiological regulator of virulence lipid anabolism (Singh et al., 2009; Cumming et al., 2017). In *M. tuberculosis*, WhiB3 directly regulated the expression of polyketide biosynthetic gene *pks2* (necessary for SL-1 production), *pks3* (necessary for PAT/DAT production), *ppsA*, *mas, fbpA* (necessary for TDM production) *fadD26* or *fadD28* (necessary for PDIM production), hence influenced the production of complex lipids (Singh et al., 2009). The MSMEG_4727 gene (*pks5*) in *M. smegmatis* was identified to be homologous to *M. tuberculosis pks2* with a 65% identity according to the KEGG database. To validate whether *pks5* was the target gene of WhiB3 in *M. smegmatis*, the EMSA assay was performed with apo-WhiB3 protein treated with diamide as described previously (Singh et al., 2009) under anaerobic conditions. As shown in Figure 5A, the result revealed a direct binding interaction, as DNA probes clearly shifted following incubation with WhiB3.

To investigate the regulatory effect of WhiB3 on *pks5*, we tried to construct the *M. smegmatis* Δ*whiB3* strains, unfortunately, we were unable to construct the deletion strain. The WT strain and WhiB3 overexpression strain (O*whiB3*) were then used for the RT-PCR experiments. The data showed that *pks5* transcript level was markedly increased (4-fold) in O*whiB3* strain (Figure 5B), indicating that WhiB3 directly activated the transcription of *pks5*. The impact of nitrogen availability on *pks5* transcription was then analyzed. We compared *pks5* transcription level under defined N^XS^ and N^L^ conditions. The result showed that transcript level of *pks5* was approximately 2-fold higher under the N^L^ condition compared with the N^XS^ condition, while in the Δ*glnR* mutant, no obvious changes in *pks5* transcription level were observed in response to nitrogen starvation. The *glnR* complementation partially restored the N^L^ activation effect as observed in WT strain (Figure 5C). These results indicated that GlnR activated *pks5* transcription during nitrogen starvation.

### GlnR Activates the Synthesis of SL-1 Lipid Through WhiB3

The SL-1 lipid was informed to play a crucial role in organization and pathogenesis of *M. tuberculosis* cell envelopes and *pks2* was demonstrated necessary for its biosynthesis (Jackson et al., 2007). We chose to evaluate the effect of GlnR and nitrogen availability on the SL-1 production using thin layer chromatography (TLC) assay and quantified the band intensities using Image J software. As shown in Figure 5D, production of SL-1 had a 31% increase in *M. smegmatis* O*whiB3* over WT strain when grown in the same condition, which confirmed that WhiB3 was a positive regulator for SL-1 production. Next, we analyzed the effect of GlnR-mediated nitrogen availability on SL-1 production. In *M. smegmatis* WT strain, the production of SL-1 showed an obvious 70% improvement in response to nitrogen starvation (Figure 5D). Since GlnR was activated during *M. smegmatis* adaptation in nitrogen starvation, the implications of these findings strongly suggested that GlnR activated the synthesis of SL-1 lipid through WhiB3, which exerted new influence of nitrogen metabolism on lipid anabolism in *M. smegmatis*.

## Discussion

The WhiB-like protein family is reported to occupy an important place in actinobacteria pathogenesis and biology. The known function of WhiB in mycobacteria is as a sensor and regulator: sensing fluctuations of the intracellular redox state to maintain redox balance, and regulating the production of inflammatory lipids including PAT, DAT, SL-1, PDIM, and TAG via a redox-dependent switching mechanism (Rohde et al., 2007; Singh et al., 2007, 2009; Cumming et al., 2017). Both of which are associated with normal cellular metabolism, especially the extensively studied carbon metabolism. However, the mechanisms associated with nitrogen metabolism have not yet been established.

Nitrogen is one of the essential elements required for bacterial growth. Bacteria must adapt to nitrogen limitation for survival. In *M. smegmatis,* the transcriptional response to nitrogen limitation is thought to be regulated by OmpR-family protein GlnR (Amon et al., 2008, 2009, 2010). At present, knowledge of the genetic response to nitrogen starvation and nitrogen metabolism in mycobacteria is still limited (Malm et al., 2009; Jessberger et al., 2013). In this article, we provided unique insight into the regulation mechanism of nitrogen regulator GlnR on WhiB3 in *M. smegmatis* and proved that GlnR bound directly to *whiB3* promoter region, exerting regulatory effect on *whiB3* transcription as a strong positive regulator. Furthermore, GlnR was demonstrated essential in the survival of *M. smegmatis* under acidic and oxidative stress.

WhiB3 was reported as a regulator of complex virulence lipid production including SL-1, PAT, PDIM, and TAG. SL-1 was suggested to play a key role in the virulence of *M. tuberculosis*. SL-I prevented fusion of phagosome and lysosome in macrophages, inhibited mitochondrial oxidative phosphorylation, modulated the cytokine secretion and oxidative responses of human neutrophils and monocytes (Jackson et al., 2007). The *pks2* expression was necessary for the synthesis of the hydroxyphthioceranic and phthioceranic acids found in SL-1 (Graham and Clark-Curtiss, 1999). In *M. smegmatis*, *pks5*, homologous to *M. tuberculosis pks2*, was verified as target gene of WhiB3 and its transcription was strongly activated by WhiB3. Consequently, the SL-1 synthesis was under control of WhiB3 and its regulatory factor GlnR. It is noteworthy that *M. smegmatis* acquire more SL-1 lipid during nitrogen starvation, revealing positive regulatory effect of GlnR on SL-1 production. It could be speculated that transcription of other WhiB3 targets such as *fbpA* (necessary for TDM production), *pks3* (necessary for PAT/DAT production), *ppsA*, *mas*, *fadD26* or *fadD28* (necessary for PDIM production) might also be regulated by GlnR-mediated nitrogen signals, which hence influenced the production of complex lipids.

**FIGURE 6:**
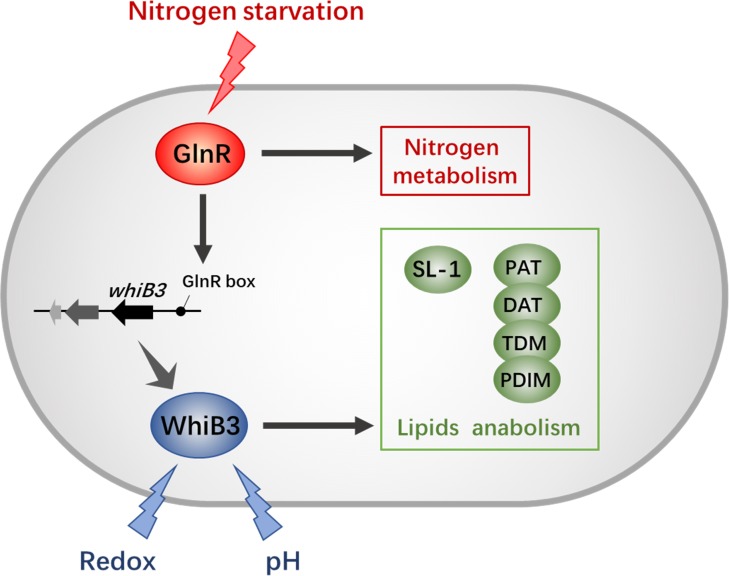
The GlnR-mediated network of redox sensing and lipid anabolism. The black solid lines with arrows indicate positive transcriptional regulation; arrows indicate the positive control. The GlnR box indicates a GlnR-binding motif.

Recent studies have shown that GlnR regulates short-chain fatty acid assimilation (Liu et al., 2018), post-translational modifications (Xu et al., 2017) and mycobacterial biofilm development (Yang et al., 2018) in mycobacteria. These facts further supported a global role of GlnR expanding beyond nitrogen assimilation and widely influencing mycobacterial adaptation. Moreover, our observation here confirmed that GlnR regulate redox sensing and lipid anabolism via transcriptional control of WhiB3. These findings suggest a tight connection between intracellular redox status, virulence and nitrogen metabolism (Figure 6). Due to the high homology of WhiB3 between *M. smegmatis* and *M. tuberculosis*, our research might serve as an enrichment of pathogen biology and the foundation for new therapeutic discovery efforts.

## Author Contributions

Ba-CY and DY designed the research. DY, YX, and Bi-CY performed the experiments. DY, YX, Bi-CY, and Ba-CY contributed to data analysis and manuscript writing. All authors contributed to manuscript revision and approved the submission.

## Conflict of Interest Statement

The authors declare that the research was conducted in the absence of any commercial or financial relationships that could be construed as a potential conflict of interest.

## References

[B1] Alam M. S. Garg S. K. Agrawal P. (2009). Studies on structural and functional divergence among seven WhiB proteins of *Mycobacterium tuberculosis* H37Rv. *FEBS J.* 276 76–93. 10.1111/j.1742-4658.2008.06755.x 19016840

[B2] Amon J. Brau T. Grimrath A. Hanbler E. Hasselt K. Holler M. (2008). Nitrogen control in *Mycobacterium smegmatis*: nitrogen-dependent expression of ammonium transport and assimilation proteins depends on the OmpR-type regulator GlnR. *J. Bacteriol.* 190 7108–7116. 10.1128/JB.00855-08 18689485 PMC2580704

[B3] Amon J. Titgemeyer F. Burkovski A. (2009). A genomic view on nitrogen metabolism and nitrogen control in mycobacteria. *J. Mol. Microbiol. Biotechnol.* 17 20–29. 10.1159/000159195 18824837

[B4] Amon J. Titgemeyer F. Burkovski A. (2010). Common patterns - unique features: nitrogen metabolism and regulation in Gram-positive bacteria. *FEMS Microbiol. Rev.* 34 588–605. 10.1111/j.1574-6976.2010.00216.x 20337720

[B5] Barry C. E. III Lee R. E. Mdluli K. Sampson A. E. Schroeder B. G. Slayden R. A. (1998). Mycolic acids: structure, biosynthesis and physiological functions. *Prog. Lipid Res.* 37 143–179. 10.1016/S0163-7827(98)00008-3 9829124

[B6] Brennan P. J. Nikaido H. (1995). The envelope of mycobacteria. *Annu. Rev. Biochem.* 64 29–63. 10.1146/annurev.bi.64.070195.000333 7574484

[B7] Chater K. F. (1972). A morphological and genetic mapping study of white colony mutants of *Streptomyces coelicolor*. *J. Gen. Microbiol.* 72 9–28. 10.1099/00221287-72-1-9 4561048

[B8] Cumming B. M. Rahman M. A. Lamprecht D. A. Rohde K. H. Saini V. Adamson J. H. (2017). *Mycobacterium tuberculosis* arrests host cycle at the G(1)/S transition to establish long term infection. *PLoS Pathog.* 13:e1006389. 10.1371/journal.ppat.1006389 28542477 PMC5456404

[B9] Daffe M. Draper P. (1998). The envelope layers of mycobacteria with reference to their pathogenicity. *Adv. Microb. Physiol.* 39 131–203. 10.1016/S0065-2911(08)60016-8 9328647

[B10] Dye C. Scheele S. Dolin P. Pathania V. Raviglione R. C. (1999). Global burden of tuberculosis - estimated incidence, prevalence, and mortality by country. WHO global surveillance and monitoring project. *JAMA* 282 677–686. 10.1001/jama.282.7.677 10517722

[B11] Flardh K. Findlay K. C. Chater K. F. (1999). Association of early sporulation genes with suggested developmental decision points in *Streptomyces coelicolor* A3(2). *Microbiology* 145(Pt 9), 2229–2243. 10.1099/00221287-145-9-2229 10517576

[B12] Gomez J. E. Bishai W. R. (2000). whmD is an essential mycobacterial gene required for proper septation and cell division. *Proc. Natl. Acad. Sci. U.S.A.* 97 8554–8559. 10.1073/pnas.140225297 10880571 PMC26986

[B13] Graham J. E. Clark-Curtiss J. E. (1999). Identification of *Mycobacterium tuberculosis* RNAs synthesized in response to phagocytosis by human macrophages by selective capture of transcribed sequences (SCOTS). *Proc. Natl. Acad. Sci. U.S.A.* 96 11554–11559. 10.1073/pnas.96.20.11554 10500215 PMC18072

[B14] Jackson M. Raynaud C. Laneelle M. A. Guilhot C. Laurent-Winter C. Ensergueix D. (1999). Inactivation of the antigen 85C gene profoundly affects the mycolate content and alters the permeability of the *Mycobacterium tuberculosis* cell envelope. *Mol. Microbiol.* 31 1573–1587. 10.1046/j.1365-2958.1999.01310.x 10200974

[B15] Jackson M. Stadthagen G. Gicquel B. (2007). Long-chain multiple methyl-branched fatty acid-containing lipids of *Mycobacterium tuberculosis*: biosynthesis, transport, regulation and biological activities. *Tuberculosis* 87 78–86. 10.1016/j.tube.2006.05.003 17030019

[B16] Jenkins V. A. Barton G. R. Robertson B. D. Williams K. J. (2013). Genome wide analysis of the complete GlnR nitrogen-response regulon in *Mycobacterium smegmatis*. *BMC Genomics* 14:301. 10.1186/1471-2164-14-301 23642041 PMC3662644

[B17] Jessberger N. Lu Y. Amon J. Titgemeyer F. Sonnewald S. Reid S. (2013). Nitrogen starvation-induced transcriptome alterations and influence of transcription regulator mutants in *Mycobacterium smegmatis*. *BMC Res. Notes* 6:482. 10.1186/1756-0500-6-482 24266988 PMC4222082

[B18] Kim T. H. Park J. S. Kim H. J. Kim Y. Kim P. Lee H. S. (2005). The whcE gene of *Corynebacterium glutamicum* is important for survival following heat and oxidative stress. *Biochem. Biophys. Res. Commun.* 337 757–764. 10.1016/j.bbrc.2005.09.115 16212936

[B19] Kolattukudy P. E. Fernandes N. D. Azad A. K. Fitzmaurice A. M. Sirakova T. D. (1997). Biochemistry and molecular genetics of cell-wall lipid biosynthesis in mycobacteria. *Mol. Microbiol.* 24 263–270. 10.1046/j.1365-2958.1997.3361705.x 9159514

[B20] Liu X. X. Shen M. J. Liu W. B. Ye B. C. (2018). GlnR-mediated regulation of short-chain fatty acid assimilation in *Mycobacterium smegmatis*. *Front. Microbiol.* 9:1311. 10.3389/fmicb.2018.01311 29988377 PMC6023979

[B21] Malm S. Tiffert Y. Micklinghoff J. Schultze S. Joost I. Weber I. (2009). The roles of the nitrate reductase NarGHJI, the nitrite reductase NirBD and the response regulator GlnR in nitrate assimilation of *Mycobacterium tuberculosis*. *Microbiology* 155 1332–1339. 10.1099/mic.0.023275-0 19332834

[B22] Manganelli R. Voskuil M. I. Schoolnik G. K. Smith I. (2001). The *Mycobacterium tuberculosis* ECF sigma factor sigmaE: role in global gene expression and survival in macrophages. *Mol. Microbiol.* 41 423–437. 10.1046/j.1365-2958.2001.02525.x 11489128

[B23] Morris R. P. Nguyen L. Gatfield J. Visconti K. Nguyen K. Schnappinger D. (2005). Ancestral antibiotic resistance in *Mycobacterium tuberculosis*. *Proc. Natl. Acad. Sci. U.S.A.* 102 12200–12205. 10.1073/pnas.0505446102 16103351 PMC1186028

[B24] Pelicic V. Jackson M. Reyrat J. M. Jacobs W. R. Jr. Gicquel B. Guilhot C. (1997). Efficient allelic exchange and transposon mutagenesis in *Mycobacterium tuberculosis*. *Proc. Natl. Acad. Sci. U.S.A.* 94 10955–10960. 10.1073/pnas.94.20.10955 9380741 PMC23543

[B25] Primm T. P. Andersen S. J. Mizrahi V. Avarbock D. Rubin H. Barry C. E. (2000). The stringent response of *Mycobacterium tuberculosis* is required for long-term survival. *J. Bacteriol.* 182 4889–4898. 10.1128/JB.182.17.4889-4898.2000 10940033 PMC111369

[B26] Raghunand T. R. Bishai W. R. (2006). Mapping essential domains of *Mycobacterium smegmatis* WhmD: insights into WhiB structure and function. *J. Bacteriol.* 188 6966–6976. 10.1128/JB.00384-06 16980499 PMC1595512

[B27] Rifat D. Bishai W. R. Karakousis P. C. (2009). Phosphate depletion: a novel trigger for *Mycobacterium tuberculosis* persistence. *J. Infect. Dis.* 200 1126–1135. 10.1086/605700 19686042

[B28] Rohde K. H. Abramovitch R. B. Russell D. G. (2007). *Mycobacterium tuberculosis* invasion of macrophages: linking bacterial gene expression to environmental cues. *Cell Host Microbe* 2 352–364. 10.1016/j.chom.2007.09.006 18005756

[B29] Singh A. Crossman D. K. Mai D. Guidry L. Voskuil M. I. Renfrow M. B. (2009). *Mycobacterium tuberculosis* WhiB3 maintains redox homeostasis by regulating virulence lipid anabolism to modulate macrophage response. *PLoS Pathog.* 5:e1000545. 10.1371/journal.ppat.1000545 19680450 PMC2718811

[B30] Singh A. Guidry L. Narasimhulu K. V. Mai D. Trombley J. Redding K. E. (2007). *Mycobacterium tuberculosis* WhiB3 responds to O-2 and nitric oxide via its [4Fe-4S] cluster and is essential for nutrient starvation survival. *Proc. Natl. Acad. Sci. U.S.A.* 104 11562–11567. 10.1073/pnas.0700490104 17609386 PMC1906726

[B31] Steyn A. J. C. Collins D. M. Hondalus M. K. Jacobs W. R. Kawakami R. P. Bloom B. R. (2002). *Mycobacterium tuberculosis* WhiB3 interacts with RpoV to affect host survival but is dispensable for in vivo growth. *Proc. Natl. Acad. Sci. U.S.A.* 99 3147–3152. 10.1073/pnas.052705399 11880648 PMC122487

[B32] Stover C. K. de la Cruz V. F. Fuerst T. R. Burlein J. E. Benson L. A. Bennett L. T. (1991). New use of BCG for recombinant vaccines. *Nature* 351 456–460. 10.1038/351456a0 1904554

[B33] Wu M. L. Gengenbacher M. Chung J. C. Chen S. L. Mollenkopf H. J. Kaufmann S. H. (2016). Developmental transcriptome of resting cell formation in *Mycobacterium smegmatis*. *BMC Genomics* 17:837. 10.1186/s12864-016-3190-4 27784279 PMC5081680

[B34] Xu Y. You D. Ye B. C. (2017). Nitrogen regulator GlnR directly controls transcription of genes encoding lysine deacetylases in actinobacteria. *Microbiology* 163 1702–1710. 10.1099/mic.0.000553 29058657

[B35] Yang Y. Richards J. P. Gundrum J. Ojha A. K. (2018). GlnR activation induces peroxide resistance in mycobacterial biofilms. *Front. Microbiol.* 9:1428. 10.3389/fmicb.2018.01428 30022971 PMC6039565

[B36] Yao L. L. Liao C. H. Huang G. Zhou Y. Rigali S. Zhang B. C. (2014). GlnR-mediated regulation of nitrogen metabolism in the actinomycete Saccharopolyspora erythraea. *Appl. Microbiol. Biotechnol.* 98 7935–7948. 10.1007/s00253-014-5878-1 24931311

[B37] You D. Wang M. M. Ye B. C. (2017). Acetyl-CoA synthetases of *Saccharopolyspora erythraea* are regulated by the nitrogen response regulator GlnR at both transcriptional and post-translational levels. *Mol. Microbiol.* 103 845–859. 10.1111/mmi.13595 27987242

[B38] You D. Yao L. L. Huang D. Escalante-Semerena J. C. Ye B. C. (2014). Acetyl coenzyme a synthetase is acetylated on multiple lysine residues by a protein acetyltransferase with a single Gcn5-Type N-acetyltransferase (GNAT) domain in *Saccharopolyspora erythraea*. *J. Bacteriol.* 196 3169–3178. 10.1128/JB.01961-14 24957627 PMC4135648

