# Supplementary material for: Nitrogen Regulator GlnR Controls Redox Sensing and Lipids Anabolism by Directly Activating the whiB3 in Mycobacterium smegmatis
Source: Front Microbiol. 2019 Jan 29;10:74. doi: 10.3389/fmicb.2019.00074 (PMC6361795; doi:10.3389/fmicb.2019.00074)
Supplement: Supplementary file 1 [file Table_1.docx]

***Supplementary Material***

**Nitrogen regulator GlnR controls redox sensing and lipids anabolism by directly activating the *whiB3* in *Mycobacterium smegmatis***

**Di You, Ying Xu, Bin-Cheng Yin, Bang-Ce Ye*******

***Correspondence:** Bang-Ce Ye: [bcye@ecust.edu.cn](mailto:bcye@ecust.edu.cn)


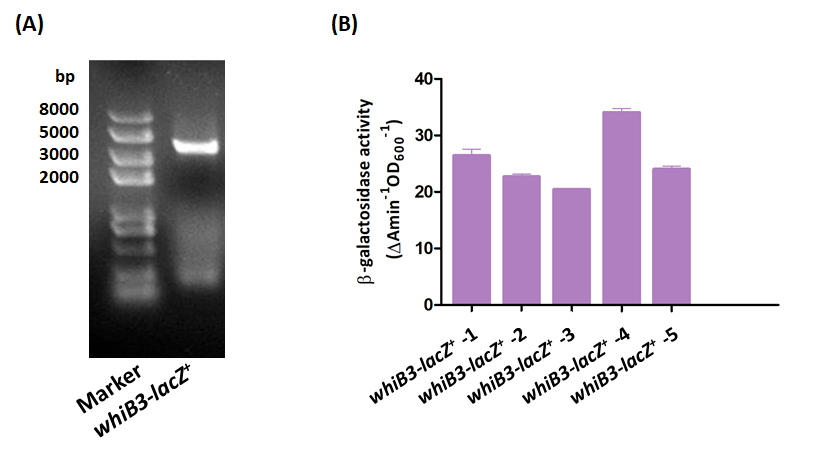


**Figure S1.** Construction of *whiB3-lacZ^+^* reporter. (A) PCR electrophoresis result of recombinant *whiB3-lacZ^+^* plasmid. (B) The β-galactosidase activities of *whiB3-lacZ^+^* reporters.

**Table S1. Strains and plasmids used in this study**

| **Strains/plasmids** | **Characteristics** | **Sources** |
| --- | --- | --- |
| **Strains** | | |
| *E. coli* DH5α | Recipient for cloning experiment | TransGen Biotech |
| BL21(DE3) | F-*ompT hsdS gal dcm* (DE3) | TransGen Biotech |
| *M. smegmatis* MC2 155 | Used as parental strain, wild type | ([Jenkins et al. 2012](#_ENREF_1)) |
| *M. smegmatis* Δ*glnR* | *M. smegmatis* *glnR* null mutant, hygromycin resistance | ([Jenkins et al. 2012](#_ENREF_1)) |
| *M.smegmatis* Δ*glnR::glnR* | *glnR* complemented strain | ([Liu et al. 2018](#_ENREF_2)) |
| DMT | For mutant strain construction | TransGen Biotech |
| **Plasmids** | | |
| pET28a | Expression vector, Kan^r^ | Novagen |
| pET-*whiB3* | pET28a derivative carrying *whiB3* | This study |
| pPR27 | Suicide plasmid | ([Mathew et al. 2005](#_ENREF_3)) |
| pPR27- *whiB3*^MU^ | *whiB3*^MU^ gene expression plasmid | This study |
| pMV261 | Extrachromosomal expression plasmid | ([Stover et al. 1991](#_ENREF_4)) |
| pMV261-*whiB3* | *whiB3* gene expression plasmid | This study |

**Table S2.** **The primers used in this study**

| Primers | Sequences (5’-3’) | Characteristics |
| --- | --- | --- |
| 5784-F | CGCGGATCCGCGTTGGATCTACTGCTA | Construction and expression of GlnR protein |
| 5784-R | CCCAAGCTTGGGTCACTGACTGGTCAA |  |
| whiB3-F | CAAATGGGTCGCGGATCCGAATTCATGCCGCAGCCGCAGCAACTA | Construction and expression of WhiB3 protein |
| whiB3-R | GTGCTCGAGTGCGGCCGCAAGCTTCTAGGCGCTGCGGCGAATTCC |  |
| G1597-F | TAATGTAGAAGTTGAATTCCACCACCACCACCACCACATGCCGCAGCCGCAGCAACTA | Overexpression of *whiB3* |
| G1597-R | TTAACTACGTCGACATCGATAAGCTTCTAGGCGCTGCGGCGAATTCC |  |
| pMV-F | GTGAGTGCTAGGTCGGGACG | Universal primer of pMV 261 |
| pMV-R | TGATGCCTGGCAGTCGA |  |
| E1597-F | AGCCAGTGGCGATAAGCGATCAGCGGAAACGGGTTAG | The primers for probes used in EMSA |
| E1597-R | AGCCAGTGGCGATAAGGCGTGCGTGAGTCGGTGGA |  |
| E-pks-F | AGCCAGTGGCGATAAGTTACGGAAAAGCGAATAAAACCCC |  |
| E-pks-R | AGCCAGTGGCGATAAGGATGATGGCAACAGGAGCAACAC |  |
| RT1597-F | CCGAATGCGGACATCTGGG | The primer for RT-PCR |
| RT1597-R | TCACCGGGCAACTGCGACA |  |
| RT-lacZ-F | GATACACTTGCTGATGCGGTGCT |  |
| RT-lacZ-R | CGGTCGGGATAGTTTTCTTGCG |  |
| RT-pks-F | TGTGTTGCTCCTGTTGCCATCAT |  |
| RT-pks-R | GGGTCGTAGTACTCCTCGGCGT |  |
| RT-MSMEG-16S-F | TGACGGTATGTGCAGAAGAAGGACC |  |
| RT-MSMEG-16S-R | CGCATTCCACCGCTACACCAG |  |
| 1597-promoter-mtu-F | CACGCACGCGTTCAGCCCCGAAATGAGCGCGCA | The primer for mutation |
| 1597-promoter-mtu-R | GGGCTGAACGCGTGCGTGAGTCGGTGGAATCAG |  |

**References**

Jenkins, V. A., B. D. Robertson and K. J. Williams (2012). Aspartate D48 is essential for the GlnR-mediated transcriptional response to nitrogen limitation in Mycobacterium smegmatis. *Fems Microbiology Letters* 330(1): 38-45.

Liu, X. X., M. J. Shen, W. B. Liu and B. C. Ye (2018). GlnR-Mediated Regulation of Short-Chain Fatty Acid Assimilation in Mycobacterium smegmatis. *Front Microbiol* 9: 1311.

Mathew, R., M. Ramakanth and D. Chatterji (2005). Deletion of the gene rpoZ, encoding the omega subunit of RNA polymerase, in Mycobacterium smegmatis results in fragmentation of the beta' subunit in the enzyme assembly. *J Bacteriol* 187(18): 6565-6570.

Stover, C. K., V. F. de la Cruz, T. R. Fuerst, J. E. Burlein, L. A. Benson, L. T. Bennett, et al. (1991). New use of BCG for recombinant vaccines. *Nature* 351(6326): 456-460.
